# Sirtuin 6 Regulates the Activation of the ATP/Purinergic Axis in Endothelial Cells

**DOI:** 10.3390/ijms24076759

**Published:** 2023-04-04

**Authors:** Cecilia Astigiano, Francesco Piacente, Maria Elena Laugieri, Andrea Benzi, Christian A. Di Buduo, Carolina P. Miguel, Debora Soncini, Michele Cea, Antonella Antonelli, Mauro Magnani, Alessandra Balduini, Antonio De Flora, Santina Bruzzone

**Affiliations:** 1 Department of Experimental Medicine, University of Genova, Viale Benedetto XV, 1, 16132 Genova, Italy; 2 Department of Molecular Medicine, University of Pavia, Via C. Forlanini 6, 27100 Pavia, Italy; 3 Department of Internal Medicine, University of Genova, Viale Benedetto XV, 6, 16132 Genova, Italy; 4 IRCCS Ospedale Policlinico San Martino, Largo R. Benzi, 16132 Genova, Italy; 5 Department of Biomolecular Sciences, University of Urbino Carlo Bo, Via Saffi 2, 61029 Urbino, Italy; 6 Department of Biomedical Engineering, Tufts University, 4 Colby Street, Medford, MA 02155, USA

**Keywords:** SIRT6, ATP, NAD^+^, inflammation, endothelium, purinergic receptors

## Abstract

Sirtuin 6 (SIRT6) is a member of the mammalian NAD^+^-dependent deac(et)ylase sirtuin family. SIRT6’s anti-inflammatory roles are emerging increasingly often in different diseases and cell types, including endothelial cells. In this study, the role of SIRT6 in pro-inflammatory conditions was investigated by engineering human umbilical vein endothelial cells to overexpress SIRT6 (SIRT6+ HUVECs). Our results showed that SIRT6 overexpression affected the levels of adhesion molecules and sustained megakaryocyte proliferation and proplatelet formation. Interestingly, the pro-inflammatory activation of the ATP/purinergic axis was reduced in SIRT6+ HUVECs. Specifically, the TNFα-induced release of ATP in the extracellular space and the increase in pannexin-1 hemichannel expression, which mediates ATP efflux, were hampered in SIRT6+ cells. Instead, NAD^+^ release and Connexin43 expression were not modified by SIRT6 levels. Moreover, the Ca^2+^ influx in response to ATP and the expression of the purinergic receptor P2X7 were decreased in SIRT6+ HUVECs. Contrary to extracellular ATP, extracellular NAD^+^ did not evoke pro-inflammatory responses in HUVECs. Instead, NAD^+^ administration reduced endothelial cell proliferation and motility and counteracted the TNFα-induced angiogenesis. Altogether, our data reinforce the view of SIRT6 activation as an anti-inflammatory approach in vascular endothelium.

## 1. Introduction

Endothelial cells (ECs) are important for the regulation of vascular hemodynamics and for permeability, coagulation, and leukocyte recruitment at sites of inflammation, where ECs interact with soluble and cell surface-bound mediators. During inflammation, ECs are exposed to inflammatory mediators, such as cytokines or proteolytic enzymes, and undergo different changes, such as an increased permeability associated with cytoskeletal reorganization and an increased expression of cellular adhesion molecules (CAM), which mediate leukocyte adhesion and extravasation, as well as megakaryocyte adhesion and maturation [1,2].

Sirtuin 6 (SIRT6), a member of the mammalian NAD^+^-dependent deac(et)ylase sirtuin family, plays key roles in different processes, including DNA repair, glucose and lipid metabolism, and inflammatory responses [3,4]. Regarding inflammation, both pro- and anti-inflammatory effects of SIRT6 have been reported in different cell types. SIRT6 anti-inflammatory effects have been linked to the repression of NF-κB and to the arrest of its signaling pathway, at various levels, in macrophages and in other cell types. On the other hand, SIRT6 has been shown to induce both the transcription and the secretion (also through post-translational modifications) of inflammatory cytokines in pancreatic cancer cells and in macrophages [4]. Recently, a critical role for SIRT6 in vascular inflammation is emerging with a consensus on SIRT6 exerting anti-inflammatory effects in vascular ECs: SIRT6 mediates the anti-inflammatory effect of hydroxytyrosol acetate, a polyphenolic compound in olive oil, and exerts a protective effect on cholesterol crystal-induced endothelial dysfunction [5,6]. Lipopolysaccharide (LPS) and tumor necrosis factorα (TNFα), two pro-inflammatory stimuli, were shown to significantly reduce the expression levels of SIRT6 in ECs [7,8]. SIRT6 silencing increased the expression of pro-inflammatory cytokines, extracellular matrix remodeling enzymes, and other pro-inflammatory pathways triggered by LPS [7], while the overexpression of SIRT6 inhibited the TNFα-induced expression of pro-inflammatory cytokines [8]. In human umbilical vein endothelial cells (HUVECs) stimulated with inflammatory cytokines, the endothelial-to-mesenchymal transition (EndMT) and the concomitant downregulation of SIRT6 expression were reported to occur with SIRT6 overexpression attenuating the EndMT process and the expression and release of pro-inflammatory cytokines (IL-6, ICAM-1). Accordingly, EndMT and the expression of pro-inflammatory cytokines were promoted in endothelium-specific SIRT6 knockout (ecSIRT6^−/−^) mice [9]. 

Pannexin-1 (PANX1) is involved both in leukocyte adhesion and migration and in ATP release from ECs [10,11]. ATP, in addition to being fundamental as an intracellular “energy” molecule, is one of the so-called damage-associated molecular pattern molecules (DAMPs) released by cells. Extracellular ATP (eATP) activates the P2 purinergic receptors, for which activation, through different mechanisms, leads to an increase in the intracellular Ca^2+^ concentration [Ca^2+^]_i_ and the subsequent regulation of cellular responses [12]. While PANX1 mediates the release of ATP, connexin43 (CX43) hemichannels are responsible for NAD^+^ transport through the plasma membrane [13]. Extracellular NAD^+^ (eNAD^+^), as well as ATP, has been reported to activate some members of the P2 receptors [14,15,16].

In this study, we aimed to comprehensively investigate the effects of SIRT6 in HUVECs stimulated with the pro-inflammatory cytokine TNFα by evaluating angiogenesis and cell adhesion, including the expression of PANX1 and CX43 and the release and effects of ATP and NAD^+^. Our results confirm an anti-inflammatory role of SIRT6 in HUVECs, which is also mediated by the reduced activation of the ATP/purinergic axis.

## 2. Results

### 2.1. SIRT6 Overexpression in Endothelial Cells Affects Leukocyte Adhesion and Megakaryocyte Function

**Figure 1:**
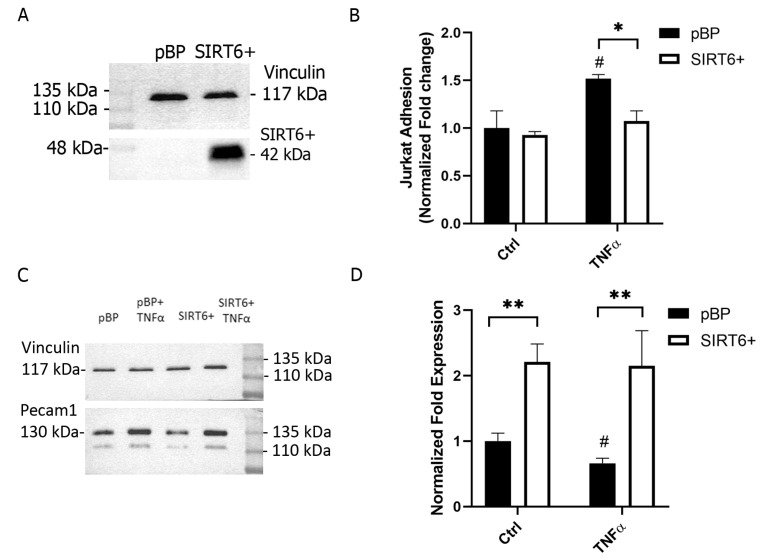
SIRT6 affects cell adhesion. (**A**) HUVECs were engineered to stably overexpress SIRT6 (SIRT6+) and SIRT6 levels were checked by Western blot analysis (pBP, cells infected with the empty plasmid). (**B**–**D**) HUVECs were treated with 10 ng/mL TNFα for 24 h. (**B**) adhesion of calcein-loaded Jurkat cells to HUVECs was evaluated with a fluorescence plate reader (means ± SD of *n* = 4 determinations are shown). (**C**,**D**) The expression of PECAM-1 was analyzed by Western blot analysis and normalized towards vinculin levels (a representative blot is shown in C and the mean ± SD of *n* = 3 analysis is shown in D). * *p* < 0.05; ** *p* < 0.01; ^#^ *p* < 0.05 vs. the respective control.

SIRT6’s role in inflammation in different contexts and cell types has been previously reported [3,4]. To investigate the possible involvement of SIRT6 in the regulation of inflammatory processes in ECs, we engineered a human endothelial cell line, HUVEC, to stably overexpress SIRT6 (SIRT6+) or the empty vector pBP (Figure 1A).

SIRT6 has been previously shown to control cell adhesion: the gene expression of the intercellular adhesion molecule (ICAM) was up-regulated in SIRT6-silenced, LPS-treated HUVECs [7], while the TNFα-induced overexpression of ICAM was abrogated in SIRT6 overexpressing cells [9]. Thus, the in vitro adhesion of leukocytes (Jurkat cells) to pBP and SIRT6+ HUVECs was assessed both in basal and in pro-inflammatory conditions: as expected, Jurkat cells’ adhesion to TNFα-treated pBP HUVECs was increased compared to the control cells (Figure 1B). Conversely, SIRT6 overexpression counteracted TNFα effect on cell adhesion (Figure 1B), confirming the role of SIRT6 in controlling leukocyte adhesion to ECs [7,17].

To evaluate whether SIRT6 also regulates the platelet-EC adhesion molecule-1 (PECAM-1), a key player in leukocyte/megakaryocyte adhesion and activation [17,18], we examined the expression levels of PECAM-1 after the treatment of HUVECs with TNFα (10 ng/mL for 24 h). SIRT6 overexpression determined an upregulation of PECAM-1 in basal conditions and prevented the TNFα-induced downregulation of PECAM-1 (Figure 1C).

**Figure 2:**
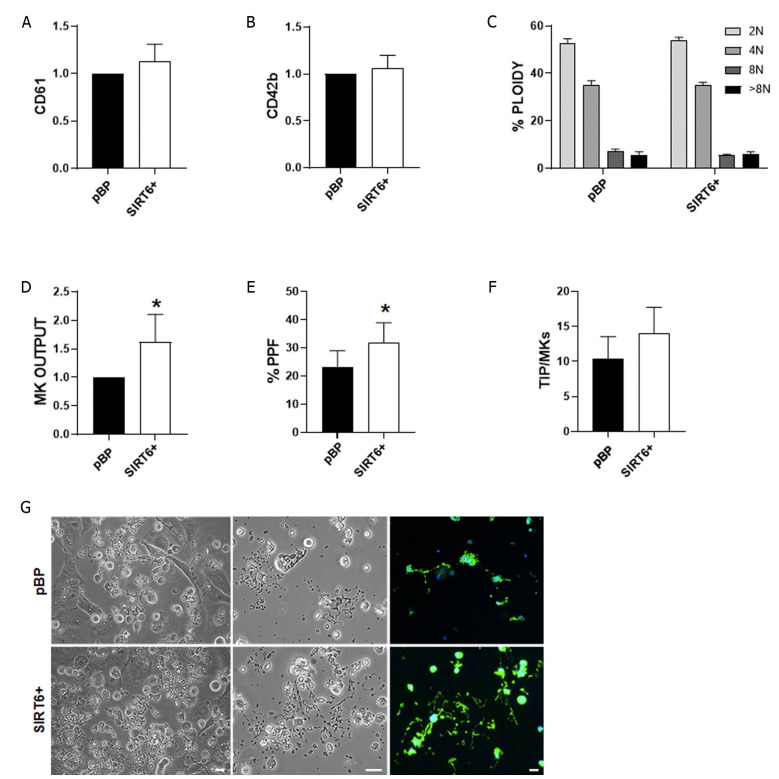
Influence of SIRT6+ HUVECs on megakaryopoiesis. (**A**,**B**) Analysis of cell surface maturity markers, CD61 (integrin β3) and glycoprotein Ib (CD42b), was performed by flow cytometry. Data are expressed as mean ± SD of the mean fluorescence intensity of megakaryocyte (MK) cocultured with SIRT6+ HUVECs with respect to pBP. (**C**) Megakaryocyte ploidy was quantified at the end of the culture by flow cytometry by gating CD61+ events within the corresponding parameters of size and complexity to mature cells. No significant differences have been observed (*p* = NS). Data are expressed as mean ± SD. (**D**) Analysis of megakaryocyte output in the different tested conditions. Output has been defined as the total number of megakaryocytes/condition at the end of the culture. Data are expressed as mean ± SD. (**E**) Percentage of proplatelet formation. (**F**) The mean number of proplatelet tips (TIP) was calculated as the number of platelet-like terminal ends/megakaryocytes (TIP/MKs). (**G**) Representative microscopy analyses of proplatelet formation after HUVEC/megakaryocyte coculture in the different tested conditions. Proplatelets have been identified as cells elongating branching filaments with platelet-sized tips at their terminal ends (phase contrast: scale bar = 20 μM; immunofluorescence: green = β1-tubulin; blue = nuclei; scale bar = 30 μM). * *p* < 0.05.

When megakaryocyte progenitors were cocultured with SIRT6+ HUVECs, we observed comparable terminal differentiation with respect to cells cocultured with pBP, as demonstrated by the analysis of the late-differentiation markers, integrin β3 (CD61) and glycoprotein Ib (CD42b) (Figure 2A,B). Physiologically, megakaryocytes undergo successive rounds of endomitosis during differentiation, resulting in polyploidy [19]. Here, we demonstrated that both conditions support efficacious polyploidization with no significant differences between the two tested conditions (Figure 2C).

Notably, in the presence of SIRT6+ HUVECs, there was a significantly increased output of mature megakaryocytes (Figure 2D), which also showed an improved ability to form and elongate proplatelet branches (Figure 2E,F), thus demonstrating that SIRT6+ HUVECs positively influence thrombopoiesis (Figure 2G).

### 2.2. SIRT6 Regulates ATP Release from HUVECs

**Figure 3:**
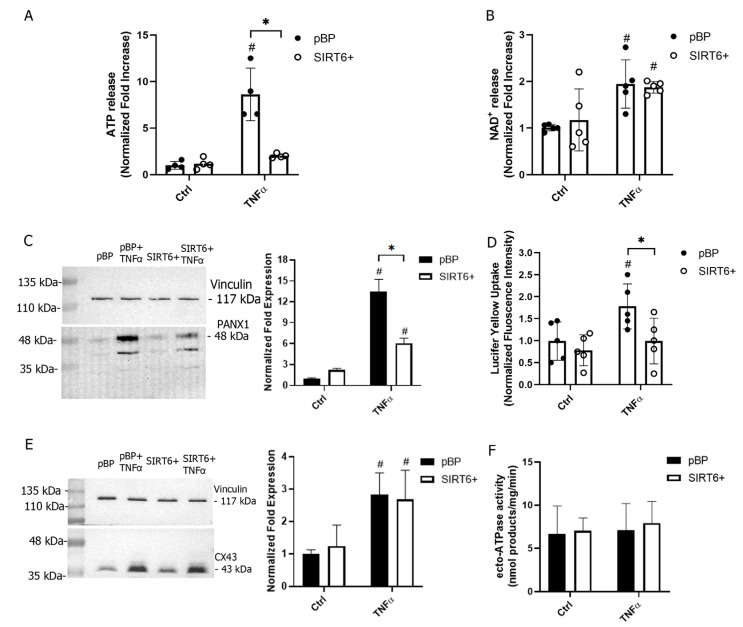
SIRT6 affects ATP, but not NAD^+^, release from HUVECs. HUVECs were stimulated (or not stimulated) with 10 ng/mL TNFα for 24 h. ATP (**A**) and NAD^+^ (**B**) releases were evaluated after 30 min incubation in HBSS (*n* = 4 for ATP and *n* = 5 for NAD^+^ release). (**C**) Western blot analysis was conducted to evaluate PANX1 expression in pBP and SIRT6+ HUVECs (a representative blot and expression quantification, normalized to vinculin levels, are shown; *n* = 3). (**D**) The uptake of the fluorescent dye lucifer yellow (LY) was evaluated in a fluorescence plate reader (ex = 390 nm; em = 520 nm; *n* = 5). (**E**) Western blot analysis was conducted to evaluate CX43 expression in pBP and SIRT6+ HUVECs (a representative blot and expression quantification, normalized to vinculin levels, are shown; *n* = 3). (**F**) Ectocellular ATPase activity was evaluated by adding 0.3 mM ATP to HUVECs either pretreated or not pretreated with TNFα and ATP degradation was determined by HPLC analysis (*n* = 4). * *p* < 0.05; ^#^ *p* < 0.05 vs. the respective control.

Inflammatory responses involve the so-called damage-associated molecular pattern molecules (DAMPs). These molecules include ATP, which, in addition to its role in cell energy, can be extracellularly released by cells. eATP activates the P2 purinergic receptors [12,20] to trigger Ca^2+^-mediated cell responses. In addition to eATP, NAD^+^ can also be released and activates purinergic-mediated signaling [13,14,15,16]. The levels of eATP and eNAD^+^ released from TNFα-stimulated HUVECs were quantified: after a 30 min incubation in HBSS buffer, eATP and eNAD^+^ levels were, respectively, 8- and 2- fold higher in pBP cells pretreated with TNFα than in control cells (Figure 3A,B). SIRT6 overexpression hampered the release of ATP, but did not hamper the release of NAD^+^, from TNFα-stimulated HUVECs (Figure 3A,B).

Given the different release of ATP in pro-inflammatory conditions from pBP and SIRT6+ cells, the expression of PANX1, mediating ATP release [21], was evaluated. The PANX1 level was greatly enhanced in TNFα-treated pBP cells (Figure 3C). SIRT6 overexpression counteracted PANX1 increase in response to TNFα (Figure 3C), in line with the unmodified levels of eATP (Figure 3A). The uptake of lucifer yellow (LY), a fluorescent dye used to evaluate hemichannel permeability, was increased in response to TNFα stimulation in pBP, but not in SIRT6+ HUVECs (Figure 3D), which is in line with the results obtained on PANX1 expression and ATP release (Figure 3A,C).

As shown in Figure 3E, the level of CX43, mediating NAD^+^ release [13], was also increased by TNFα, although to a lower extent than PANX1 (approximately 3- vs. 12-fold for CX43 and PANX1, respectively). The TNFα-induced expression of CX43 was not significantly different in SIRT6+ than in pBP cells (Figure 3E), which is consistent with eNAD^+^ levels.

To verify whether the absence of eATP increase in SIRT6+ cells was due to enhanced levels of ATPase ectoenzymatic activity expressed on the plasma membrane, and, thus, to an increased eATP degradation, intact HUVECs were incubated with exogenously added ATP (0.3 mM) and its degradation was evaluated. As shown in Figure 3F, the ecto-ATPase activity, both in basal and in TNFα-stimulated conditions, was not different in pBP or in SIRT6+ cells, suggesting that the expression of ecto-ATPases is not profoundly affected.

Altogether, these results indicate that the anti-inflammatory effects exerted by SIRT6 may also be due to the dampening of ATP release from HUVECs.

### 2.3. SIRT6 Affects the Response to eATP in HUVECs

**Figure 4:**
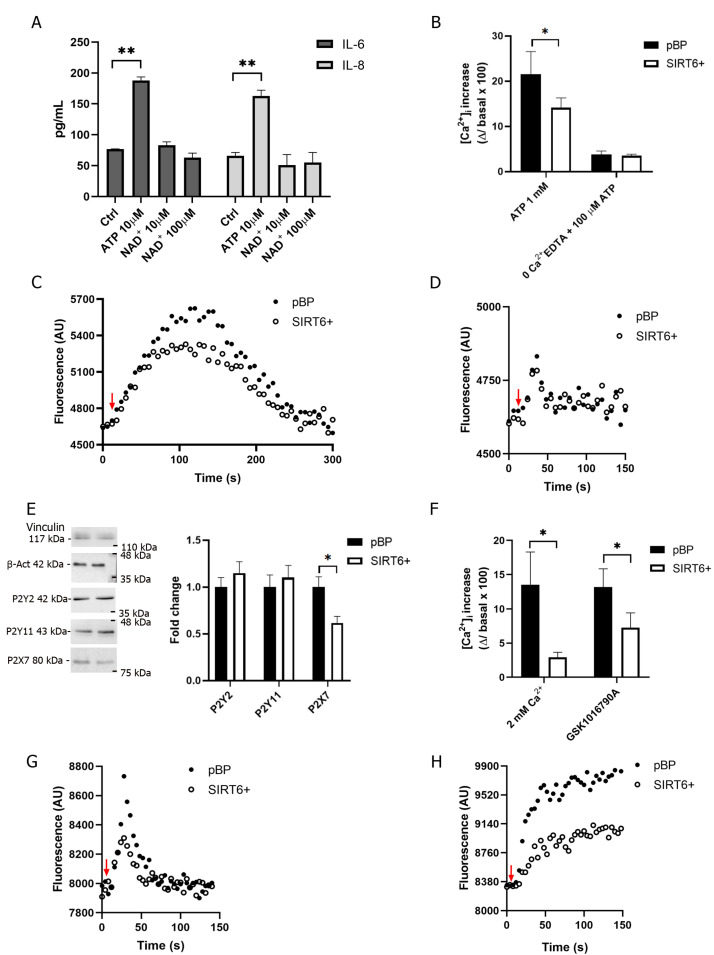
SIRT6 affects ATP-dependent Ca^2+^ response in HUVECs. (**A**) pBP cells were stimulated with 10 μM ATP or with 10 or 100 μM NAD^+^ for 6 h. IL-6 and IL-8 levels in the supernatants were evaluated with commercially available ELISA assays (*n* = 3). (**B**–**D**) pBP and SIRT6+ HUVECs were treated with 10 ng/mL TNFα for 24 h. Cells were loaded with Fluo-3 and fluorescence changes were recorded with a fluorescence plate reader. HUVECs were stimulated with 1 mM ATP in HBSS (**B**,**C**) or with 100 μM ATP in Ca^2+^-free HBSS (**B**,**D**); means ± SD of at least *n* = 8 determinations are presented in (**B**) and representative traces are shown in (**C**,**D**). (**E**) pBP and SIRT6+ HUVECs were treated with 10 ng/mL TNFα for 24 h and Western blot analyses were performed to evaluate P2Y11, P2Y2, and P2X7 expression, normalized to vinculin (a representative blot and expression quantification, normalized to vinculin and β-actin levels, are shown; *n* = 3). (**F**–**H**) pBP and SIRT6+ HUVECs were treated with 10 ng/mL TNFα for 24 h. Next, Fluo-3-loaded cells were incubated in Ca^2+^-free HBSS, prior to addition of 2 mM Ca^2+^ (**F**,**G**), and stimulated with 50 nM GSK1016790A (a TRPV4 agonist) in HBSS (**F,H**); means ± SD of at least *n* = 4 determinations are shown in (**F**) and representative traces are shown in (**G**,**H**). In (**B**,**F**), Ca^2+^ changes (Δ = difference between intensity at peak upon addition and basal intensity) were calculated for each trace using the formula Δ/basal × 100. Red arrows indicate the addition of the stimulus. * *p* < 0.05; ** *p* < 0.01.

To confirm the pro-inflammatory role of eATP on HUVECs, exogenous ATP was added: eATP triggered the release of IL-6 and IL-8 (Figure 4A). Conversely, eNAD^+^ failed to stimulate IL-6 and IL-8 secretion (Figure 4A), suggesting that eNAD^+^ exerts different effects than eATP.

To further investigate whether the response to ATP is affected by SIRT6 overexpression, the ATP-induced Ca^2+^ increase was compared in SIRT6+ and in pBP cells pretreated with TNFα for 24 h. The ATP-sensitive purinergic receptor P2 family is divided into the subfamilies of metabotropic P2Y receptors, evoking Ca^2+^ release from intracellular stores, and ionotropic P2X receptors, evoking Ca^2+^ influx from the extracellular space [12,20]. The Ca^2+^ increase induced by 1 mM ATP in the presence of extracellular Ca^2+^ (the condition to activate all the members of the P2 family receptors, including P2X7) was reduced in SIRT6+ cells (Figure 4B,C). In the absence of extracellular Ca^2+^, the response to eATP was greatly reduced, both in pBP and in SIRT6+, to similar levels (Figure 4B,D), indicating that receptors of the P2X family are mainly involved in the ATP-induced response and that the P2Y receptor(s) activation (responsible for the response to 100 μM ATP added in the absence of extracellular Ca^2+^, as shown in Figure 4B,D) is similar in the two cell types. Among the members of the P2Y subfamily, P2Y2 and P2Y11 are involved in the response to ATP in HUVECs [22,23]: the expression level of P2Y2 and P2Y11 was similar in pBP and SIRT6+ cells (Figure 4E), which is consistent with the ATP-induced Ca^2+^ release from intracellular stores (Figure 4D). Interestingly, P2X7 expression was higher in pBP than in SIRT6+ cells (Figure 4E), suggesting that the more pronounced extracellular Ca^2+^ influx (Figure 4C) is mediated by this purinoceptor.

The fact that in the absence of extracellular Ca^2+^ the response to ATP was smaller than in the presence of extracellular Ca^2+^, may suggest that, in addition to P2X7, store-operated Ca^2+^ entry (SOCE) mechanisms also contribute to the global Ca^2+^ response. Indeed, SOCE is activated upon Ca^2+^ release from intracellular stores and, in our conditions, the overall extracellular Ca^2+^ influx in response to ATP was different in pBP and SIRT6+ cells (Figure 4C), which is possibly also a result of a contribution from a SOCE mechanism. When 2 mM Ca^2+^ was added to cells incubated in the absence of extracellular Ca^2+^, the Ca^2+^ influx was significantly smaller in SIRT6+ cells (Figure 4F,G), suggesting that a mechanism mediating Ca^2+^ influx is less active/expressed. TRPV4 was also reported to be involved in SOCE in HUVECs [24]: when pBP and SIRT6+ cells were challenged with the specific TRPV4 agonist GSK1016790A [25], the [Ca^2+^]_i_ rise was significantly smaller in SIRT6+ compared to in pBP cells (Figure 4F,H).

Thus, along with a reduced ATP release, the response to ATP is hampered by SIRT6 overexpression and mechanism(s) mediating extracellular Ca^2+^ influx are dampened in SIRT6+ cells.

### 2.4. eNAD^+^ Decreases HUVEC Proliferation and Counteracts TNFα-Induced Angiogenesis

**Figure 5:**
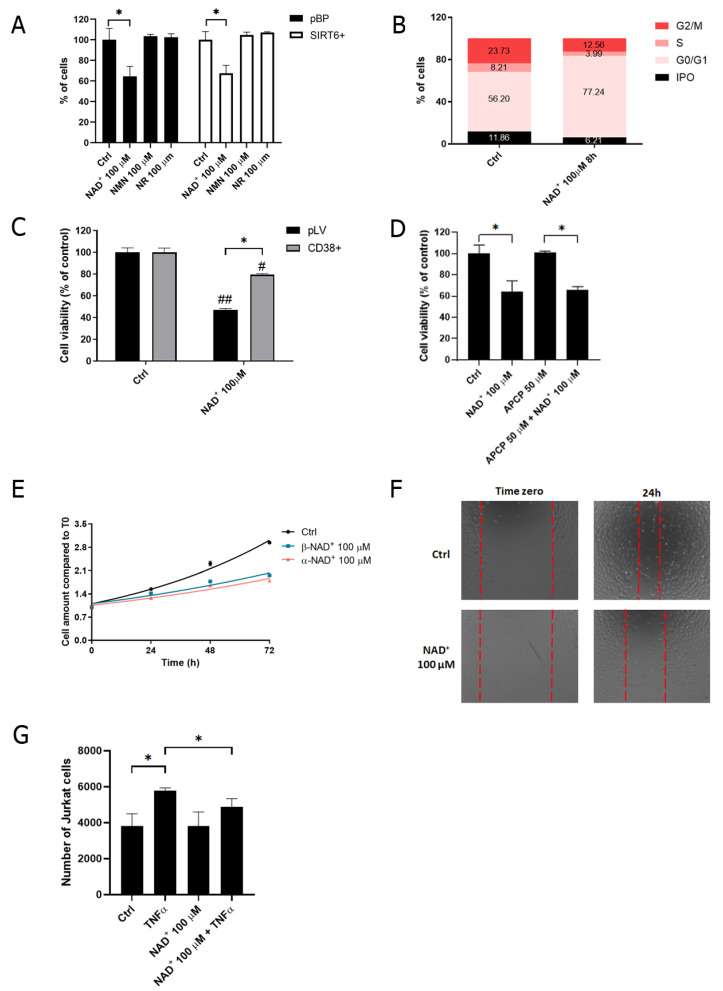
eNAD^+^ decreases HUVECs proliferation. (**A**) pBP and SIRT6+ HUVECs were treated with 100 μM NAD^+^, either nicotinamide riboside (NR) or nicotinamide mononucleotide (NMN), for 48 h. Cell viability was determined by sulforhodamine B (SRB) assay and the absorbance was measured in a plate reader (*n* = 6). (**B**) pBP and SIRT6+ HUVECs were treated with 100 μM NAD^+^ for 8 h. Cell cycle analysis was performed by FACS Calibur after cell staining with 7-Aminoactinomycin D. Cell fraction amount was determined by the peak areas in FL3 channel (*n* = 3). (**C**) HUVECs were engineered to stably overexpress CD38 (CD38+; pLV, cells infected with the empty plasmid) and exposed to 100 μM NAD^+^ for 48 h and cell viability was evaluated by SRB assay (*n* = 3). (**D**,**E**) HUVECs were treated with (**D**) 100 μM NAD^+^ in the presence or absence of 50 μM adenosine 5′-(α,β-methylene)diphosphate (APCP) for 48 h (*n* = 4) and (**E**) 100 μM α-NAD^+^ and β-NAD^+^ for 28, 48, or 72 h; cell viability was evaluated by SRB assay (*n* = 4). (**F**) HUVECs were seeded in 24-well plates and cells were treated (or not treated) with 100 μM NAD^+^: a scratch was performed with a 200-μL tip and the ability of the cells to heal the scratch was evaluated after 24 h under a microscope. A representative result is shown. (**G**) HUVECs were stimulated with 10 ng/mL TNFα in the presence or absence of 100 μM NAD^+^. After 24 h, Jurkat cells were loaded with Calcein and their adhesion to HUVECs was evaluated with a fluorescence plate reader (*n* = 4). Results are presented as mean ± SD. * *p* < 0.05; ^#^ *p* < 0.05 and ^##^ *p* < 0.01 vs. the respective control.

As shown in Figure 4, eNAD^+^ failed to stimulate IL-6 and IL-8 secretion, suggesting that eNAD^+^ exerts different (non-pro-inflammatory) effects than eATP. Thus, we subsequently aimed to evaluate the effects of eNAD^+^ on HUVECs. First, we observed that the addition of eNAD^+^ (100 μM for 48 h), but not of nicotinamide riboside or nicotinamide mononucleotide, reduced cell viability by approximately 40% in both SIRT6+ and pBP cells (Figure 5A). To determine if the decrease in cell viability was due to a decrease in cell growth or to an increase in cell death, we performed a cell cycle analysis. eNAD^+^ led to an increase in G0/G1 cell fraction, suggesting that there is a slow-down of the cell cycle, rather than increased cell death, in the presence of eNAD^+^ (Figure 5B).

eNAD^+^ is degraded by the ectoenzymes CD38 and CD73, although at a much lesser extent. HUVECs did not express CD38: from an ectoenzymatic activity analysis, the generation of ADP-ribose and nicotinamide from eNAD^+^ was not detectable. Instead, CD73 was expressed: AMP was rapidly degraded to adenosine (28.5 nmol adenosine/min/mg protein) and NAD^+^ was converted to adenosine (0.5 nmol adenosine/min/mg protein). To evaluate whether eNAD^+^ effects on cell viability are due to eNAD^+^ itself or to products of its degradation, CD38 was overexpressed in HUVECs (CD38+) to completely remove eNAD^+^ from the cell media. As shown in Figure 5C, CD38+ cell viability was significantly less affected by eNAD^+^, ruling out the possibility that this effect is mediated by CD38-produced metabolites. Instead, the presence of the intact NAD^+^ molecule seemed to affect cell viability. In addition, the pharmacological inhibition of CD73, by the use of 50 μM adenosine 5′-(α,β-methylene)diphosphate (APCP), did not reduce eNAD^+^ effects on cell viability (Figure 5D), suggesting that CD73 is not involved in the response to eNAD^+^. Given that the extracellular conversion of eNAD^+^ by ectoenzymes is not necessary to determine eNAD^+^ effects on cell viability, α-NAD^+^ was extracellularly added, since both α-NAD^+^ and β-NAD^+^ have been reported to activate P2Y11 receptors [14]. Indeed, both α-NAD^+^ and β-NAD^+^ were able to slow down the cell growth of HUVECs (Figure 5E). Moreover, treatment with eNAD^+^ decreased the ability of HUVECs to heal the scratch in 24 h (Figure 5F), which could be explained by both a decrease in cell growth and cell motility.

Next, we aimed to understand if eNAD^+^ can interfere with cell adhesion both in basal and TNFα-stimulated conditions: eNAD^+^ decreased the extent of Jurkat adhesion to HUVECs in an inflammatory environment (Figure 5G).

**Figure 6:**
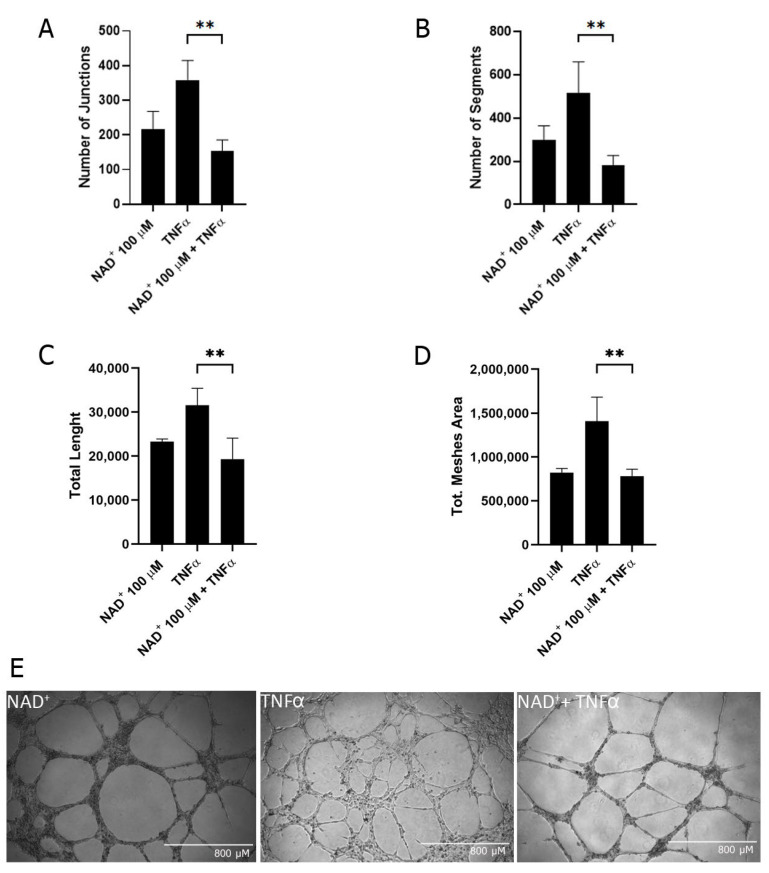
eNAD^+^ affects angiogenesis in TNFα-stimulated HUVECs. HUVECs were seeded in 96-well plates precoated with 75 μL of Matrigel. Cells were stimulated (or not stimulated) with 10 ng/mL TNFα in the presence or absence of 100 μM NAD^+^. After 8 h of incubation, pictures were taken. Different parameters to evaluate tube formation were acquired: number of junctions (**A**), number of segments (**B**), total length (**C**), and total mesh area (**D**). Results are presented as mean ± SD of values from 3 pictures/well from at least 3 wells/condition. (**E**) Representative images are presented. ** *p* < 0.01.

During inflammation, angiogenesis is stimulated in ECs [26]. Given our results regarding the role of eNAD^+^ in HUVECs motility, we investigated whether eNAD^+^ could affect tube formation in pro-inflammatory conditions. HUVECs were treated (or not treated) with TNFα (10 ng/mL for 8 h) in the presence or absence of eNAD^+^, and the number of junctions, total length, number of segments, and total mesh areas were evaluated (Figure 6). As a result, TNFα-induced angiogenesis was significantly counteracted by the administration of eNAD^+^.

## 3. Discussion

ECs play a critical role in inflammation by responding to different endogenous and exogenous pro-inflammatory stimuli. A role for SIRT6 in counteracting inflammation in ECs has clearly emerged in recent years. In a first study exploring SIRT6 role in HUVECs, LPS was administered to simulate endothelial inflammation in a context of sepsis: SIRT6 silencing was accompanied by the increased expression of pro-inflammatory cytokines, extracellular matrix remodeling enzymes, the adhesion molecule ICAM-1, and proangiogenic growth factors VEGF and FGF-2 [7]. In addition, SIRT6 silencing increased cell migration and leukocyte adhesion [7]. The TNFα-induced monocyte adhesion to SIRT6-overexpressing HUVECs was reduced because of a decrease in the TNFα-induced expression of VCAM-1 in ECs, suggesting that SIRT6 may mediate an atheroprotective effect [27]. Moreover, SIRT6 overexpression decreased the levels of multiple genes involved in vascular inflammation and atherosclerosis, such as VCAM-1, ICAM-1, and platelet selectin (P-selectin), leading to the reduced infiltration of macrophages and foam cells [28], and inhibited TNFα-induced inflammation in HUVECs regulating the Nrf2 signalling pathway [8,27]. More recently, by using endothelium-specific SIRT6 knockout mice and HUVECs, SIRT6 was demonstrated to inhibit the endothelial-to-mesenchymal transition by attenuating the vascular endothelial inflammatory response [9]. Finally, SIRT6 silencing was demonstrated to activate pro-inflammatory pathways (VCAM-1, COX-2, TNFα) in ECs [29].

The investigation of SIRT6’s role in TNFα-stimulated conditions allowed us to confirm the anti-inflammatory effects obtained by SIRT6 overexpression in HUVECs: regulating leukocyte adhesion, as well as PECAM-1 expression (Figure 1). In our study, an interesting link between SIRT6 and the PANX1/ATP/purinergic receptor axis was unveiled. PANX1 was demonstrated to be crucial for leukocyte adhesion and extravasation with its role in mediating the TNFα-induced ATP release [10]. We demonstrated that SIRT6 regulates both PANX1 expression and ATP release in response to TNFα, whereas the enzyme degrading eATP was not modulated, neither in inflammatory conditions nor by SIRT6 overexpression (Figure 3). Mechanisms to boost CD39 activity would represent useful approaches to diminish the ATP/purinergic receptor response activated in inflammation [30,31]. Indeed, the ATP-mediated purinergic receptor activation represents a pro-inflammatory stimulus in ECs [32,33]. Our results point to a decreased sensitivity to ATP in SIRT6+ cells in terms of a reduced Ca^2+^ influx (Figure 4C), which is possibly related to a reduced expression of the purinergic receptor P2X7 (Figure 4E). Instead, the ATP-induced activation of the member of the P2Y subfamily does not seem to be affected by SIRT6 expression (Figure 4D). In addition to the attenuated P2X activation, our results also indicate that the expression of SIRT6 may reduce multiple mechanisms mediating Ca^2+^ influx. Indeed, the TRPV4-dependent [Ca^2+^]_i_ increase is affected in SIRT6+ cells (Figure 4H). Interestingly, the activation of TRPV4 has been reported to occur downstream of a TNFα-induced PANX1 activation [10,34]; thus, it would be possible to conceive that an overall hampered response to TNFα in SIRT6+ cells would be due to reduced PANX1 expression and ATP release (Figure 3A,C), and possibly also concomitantly halted by TRPV4 activation. A link between SIRT6 and Ca^2+^ homeostasis, involving TRPM2 channels, had been previously demonstrated in pancreatic cancer cells [35], but a correlation between SIRT6 and the ATP/P2X/Ca^2+^ pathway has not been highlighted yet.

In granulocytes and monocytes, eNAD^+^ exerted similar pro-inflammatory responses to eATP via the activation of members of the P2Y subfamily [14,36,37,38]. Nevertheless, eNAD^+^ has also been reported to suppress the ATP-induced release of IL-1β by monocytic cells [39], indicating that different roles may be played by eNAD^+^. In HUVECs, eNAD^+^ failed to induce the release of cytokines that are, instead, secreted in response to ATP stimulation (Figure 4A). This result suggested that eNAD^+^ may play different roles than ATP in different cell types. Indeed, NAD^+^ administration had been previously demonstrated to attenuate the IL-1β-induced inflammatory response in HUVECs, reducing the expression of adhesion molecules (VCAM-1 and ICAM-1) and MCP-1. Moreover, NAD^+^ administration in mice could alleviate vascular endothelial dysfunction in the aorta [40]. Along this line of evidence, NAD^+^ has been demonstrated to be released by ECs in response to LPS and to counteract the LPS-induced damage by controlling vascular permeability, maintaining a restrictive endothelial barrier, and increasing the surface area of cell–cell interfaces [41]. Overall, our results are in agreement with an anti-inflammatory role exerted by eNAD^+^ in ECs because it (a) reduced cell motility and proliferation (Figure 5); (b) attenuated the TNFα-induced leukocyte adhesion to HUVECs (Figure 5G); and (c) abrogated the TNFα-induced angiogenesis (Figure 6). Nevertheless, it should be underlined that in non-inflammatory conditions, NAD^+^ administration may evoke completely different effects: in isolated embryonic periventricular ECs, NAD^+^ enhanced proliferation and angiogenesis in the absence of Matrigel [42].

This study highlighted that SIRT6+ HUVECs can better sustain megakaryocyte function, as demonstrated by the improved ability to form and elongate proplatelet branches (Figure 2), which assemble platelets at their terminal ends [43]. This effect is in agreement with previous evidence demonstrating that megakaryocyte coculture with different types of stromal cells, including HUVECs, positively influences cell proliferation, maturation, and proplatelet formation [44]. The positive effect of SIRT6 expression in HUVECs may be related to the increased expression of PECAM-1 (also known as CD31; Figure 1C), a fundamental mediator of megakaryocyte–EC interaction [45]. The recovery of the peripheral platelet count is impaired in PECAM-1^−/−^ mice following experimentally induced thrombocytopenia [18], which is likely due to a reduced megakaryocyte migration towards endothelium. Thus, it is conceivable that the increased expression of PECAM-1 in SIRT6+ ECs could ameliorate the contacts between the two cell types, leading to an improved stimulus to make platelets.

## 4. Materials and Methods

### 4.1. Cell Culture and Differentiation

Cryopreserved HUVECs were purchased from Lonza (Walkersville, MD, USA). HUVECs were cultured in Endothelial Growth Medium-2 (EGM-2) (Lonza, Basel, Switzerland) in a humidified atmosphere with 5% CO_2_ at 37 °C. HUVECs were engineered to stably overexpress Sirtuin 6 (SIRT6+) or the corresponding empty plasmid (pBP) by retroviral transduction.

HUVECs were engineered to stably overexpress CD38 (CD38+) or the corresponding empty plasmid (pLV) by lentiviral transduction.

Jurkat cells were purchased from ATCC (LGC Standards, Italy) and were cultured in RPMI-1640 (Euroclone) with 10% FBS in a humidified atmosphere with 5% CO_2_ at 37 °C.

Megakaryocytes were differentiated from human CD34+ hematopoietic stem and progenitor cells from umbilical cord blood using previously described methods [46]. Human umbilical cord blood was collected following normal pregnancies upon informed consent of the parents under the ethical committee of the IRCCS Policlinico San Matteo Foundation of Pavia and in accordance with the principles of the Declaration of Helsinki. CD34+ cells were separated by an immunomagnetic column and cultured for two weeks in Stem Span medium (STEMCELL Technologies, Vancouver, Canada) supplemented with 10 ng/mL thrombopoietin (TPO), 10 ng/mL IL-11, 1% L-glutamine, and 1% penicillin-streptomycin. SIRT6+ and pBP HUVECs were seeded at a density of 2 × 10^4^/cm^2^ in 6-well plates and left to grow until confluence before starting megakaryocyte/endothelial cell coculture.

### 4.2. Analysis of Proplatelet Formation

Megakaryocyte output and proplatelet yields were evaluated at the end of the cell culture as previously described [46]. Proplatelets were identified as cells displaying long filamentous structures with platelet-sized tips. The percentage of megakaryocytes bearing proplatelets was determined by analyzing at least 20 random fields. Phase contrast images were obtained by an Olympus IX53 microscope (Olympus, Tokyo, Japan). For immunofluorescence analysis, cells were fixed in 4% paraformaldehyde (Sigma-Aldrich, St. Louis, MO, USA), permeabilized with 0.5% Triton X-100 (Sigma-Aldrich), blocked with 5% BSA, and stained with anti-β1-tubulin antibody (Abcam, Cambridge, UK, 1:1000) for 1 h at room temperature and Alexa Fluor secondary antibody (Invitrogen, Waltham, MA, USA, 1:500). Nuclear counterstaining was performed using Hoechst 33258 (100 ng/mL, Sigma-Aldrich). Specimens were mounted in ProLong Gold Antifade reagent (Invitrogen, Waltham, MA, USA). Negative controls were routinely performed by omitting the primary antibody. Immunofluorescence images were acquired by a Nikon Eclipse Ti2 microscope (Olympus). Megakaryocytes extending proplatelets were identified as cells extending tubulin-positive long filamentous structures ending with platelet-sized tips.

### 4.3. Flow Cytometry

Differentiation parameters of cultured megakaryocytes were analyzed by flow cytometry. Samples were stained with CD61-FITC and CD42b-PE antibodies (Abcam, Cambridge, UK). For megakaryocyte ploidy, cells were fixed overnight in ice-cold 70% ethanol at −20 °C. Samples were then incubated in 100 μg/mL of RNAse and propidium iodide solution and stained with antihuman CD61-FITC antibody. All samples were acquired with a BD FACSLyric™ Flow Cytometry System. Offline data analysis was performed using the Beckman Coulter Kaluza software package (Brea, CA, USA).

### 4.4. Release of IL-6 and IL-8

HUVECs (5 × 10^4^ cells) were seeded in 24-well plates. The day after, cell culture medium was removed, and cells were rinsed with HBSS (Hank’s balanced salt solution) and then incubated in HBSS for 6 h at 37 °C in the presence or absence of ATP and NAD^+^ (final concentration 100 μM). IL-8 and IL-6 releases in the supernatant were evaluated using commercial ELISA kits (Biolegend, San Diego, CA, USA).

### 4.5. Western Blot Analyses

HUVECs were lysed in 25 mM Tris-HCl, pH 7.8, 2 mM DTT, 2 mM EDTA, 10% glycerol, and 1% Triton X-100, with the addition of protease inhibitor (Complete mini, Roche, Basel, Switzerland) and phosphatase inhibitor (Sigma Aldrich, St. Louis, MO, USA). Lysates (20 μg proteins) were loaded on a 10% polyacrylamide gel and proteins were separated by SDS-PAGE and transferred to nitrocellulose membranes. Detection was performed with the following primary antibodies (Table 1):

Following incubation with the appropriate secondary antibodies and ECL detection (GE Healthcare, Milan, Italy), band intensity was quantified with the ChemiDoc imaging system (Bio-Rad, Milan, Italy).

### 4.6. Angiogenesis Assay

HUVECs (2 × 10^4^ cells) were seeded in 96-well plates precoated with Matrigel (75 μL/well) (Corning, One Riverfront Plaza, Corning, NY, USA), which polymerized upon incubation at 37 °C for 30 min. TNFα-treated (10 ng/mL, 8 h) or control cells were incubated in the presence or absence of 100 μM NAD^+^. After 8 h, images were taken with the microscope EVOS M5000 (4× objective). Pictures were analyzed using a plug-in studied for ImageJ: Angiogenesis Analyzer [47].

### 4.7. Adhesion of Jurkat Cells to HUVECs

HUVECs (4 × 10^4^ cells/well) were seeded in 96-well plates and cultured for 24 h. HUVECs were stimulated, or not stimulated, with 10 ng/mL TNFα for 24 h. Jurkat cells were loaded with 10 μM Calcein for 1 h at 37 °C. After the labelling, Jurkat cells were washed with DPBS (Dulbecco′s Phosphate Buffered Saline), resuspended in RPMI-1640, and added (5 × 10^4^ cells/well) to the ECs after a wash-out of the EGM-2 medium with HBSS.

Jurkat and HUVECs were coincubated for 1 h at 37 °C. After the incubation, cells were washed three times with DPBS and 100 μL of DPBS was added in each well and fluorescence intensity was measured in a micro-plate reader (Fluostar Optima; BMG Labtechnologies, Offenburg, Germany).

### 4.8. Release of Extracellular ATP and NAD^+^

Releases of eATP and eNAD^+^ were evaluated after a pro-inflammatory stimulus (10 ng/mL TNFα for 24 h). After collection and centrifugation, supernatants were assayed for evaluation of eATP levels by a luciferin–luciferase assay (ATP Bioluminescence Assay Kit CLS II; Roche, Basel, Switzerland) and of eNAD^+^ levels by an enzymatic cycling assay using a micro-plate reader. Protein determination was performed on cell lysates by Bradford assay.

### 4.9. Viability Assay

HUVECs (2 × 10^3^ cells) were seeded in 96-well plates. After 24 h, cells were treated, or not treated, with 100 μM NAD^+^, NR, or NMN. After 48 h, cell density was determined by sulforhodamine B (SRB) assay by measuring the absorbance at 564 nm [48].

### 4.10. Wound-Healing Assay

HUVECs were cultured upon reaching confluence in 24-well plates. Cells were scratched with the head of a 200-μL tip and they were treated with 100 μM NAD^+^ [49]. The ability of the cells to heal the scratch was imaged under a Leica microscope (10X objective) at 0 and 24 h after wounding.

### 4.11. Cell Cycle Analysis

HUVECs were cultured in 6-well plates in the exponential growth phase. Cells were synchronized with 1 µM Palbociclib for 16 h. The day after, Palbociclib was removed and cells were treated, or not treated, with 100 µM NAD^+^ for 8 h. Then, cells were trypsinized, fixed, and permeabilized with alcohol and stained with 7-aminoactinomycin D. The analysis was performed with a FACS Calibur on FL3 channel.

### 4.12. Assays of Ectocellular Enzymatic Activities

HUVECs were grown in 24-well plates and the activities were measured as previously described [50,51]. After removal of the culture medium, cells were washed twice with 1 mL HBSS, and 0.35 mL HBSS containing 0.3 mM of ATP, NAD^+^, or AMP were added. At different time points (0, 5, and 10 min), aliquots of the incubation mixtures were withdrawn, and trichloroacetic acid was added (5% *v*/*v* final concentration). ATP, NAD^+^, and AMP degradation were determined by high-performance liquid chromatography (phosphate analysis) [50]. Cells were lysed and protein content in each well was determined by Bradford assay (Bio-Rad).

### 4.13. Fluorimetric Determination of Intracellular Calcium Levels

HUVECs (∼50% confluence) were cultured in 96-well plates and fluorimetric determinations to evaluate changes in the [Ca^2+^]_i_ were performed as previously described [51]. HUVECs were loaded with 10 μM Fluo-3 for 45 min at 37 °C in the complete medium and washed twice with 200 μL Ca^2+^-containing HBSS or Ca^2+^-free HBSS. Fluorescence (excitation, 485 nm; emission, 520 nm) was measured every 3 s with a fluorescence plate reader (FLUOstar Optima, BMG LABTECH, Ortenberg, Germany). The intensity of emitted light was plotted as a function of time. Calcium changes (Δ = difference between intensity at peak on addition and basal intensity) were calculated for each trace using the formula Δ/basal × 100.

### 4.14. Statistical Analysis

Statistical analyses were performed using GraphPad Prism Software (San Diego, CA, USA). Differences among groups were tested with unpaired Student’s *t*-test. A statistically significant difference was achieved for *p*-values < 0.05.

## 5. Conclusions

Functional alterations of ECs occur in many inflammatory conditions, including diabetes, hypertension, atherosclerosis, rheumatoid arthritis, and aging. Thus, understanding the players governing the response of ECs in inflammatory states is crucial to develop aids to maintain endothelial homeostasis and the prevention of vascular diseases. Very recently, we also demonstrated that SIRT6 may prevent oxidative stress damages via the upregulation of NADPH to counteract reactive oxygen species in HUVECs [52]. Altogether, our results suggest that SIRT6 activation through pharmacological approaches may represent a useful strategy to (a) counteract endothelial inflammatory responses and (b) sustain the production of platelets. In addition, we unveiled that anti-inflammatory effects may be obtained by NAD^+^ administration.

## Figures and Tables

**Table 1:** Antibodies used for Western Blot analysis.

Antibody	Company
Anti-PECAM-1	Santa Cruz Biotechnology, Inc., Dallas, TX, USA
Anti-Vinculin	Cell Signaling Technology, Danvers, MA
Anti-P2Y2	Biorbyt Ltd., Cambridge, UK
Anti-P2Y11	Alomone Labs, Jerusalem, Israel
Anti-P2X7	Alomone Labs, Jerusalem, Israel
Anti-pannexin-1	Santa Cruz Biotechnology, Inc., Dallas, TX, USA
Anti-connexin43	Santa Cruz Biotechnology, Inc., Dallas, TX, USA

## Data Availability

Not applicable.
